# Rapid microstructural plasticity in the cortical semantic network following a short language learning session

**DOI:** 10.1371/journal.pbio.3001290

**Published:** 2021-06-14

**Authors:** Nikola Vukovic, Brian Hansen, Torben Ellegaard Lund, Sune Jespersen, Yury Shtyrov

**Affiliations:** 1 Department of Psychiatry, University of California San Francisco, San Francisco, United States of America; 2 Center of Functionally Integrative Neuroscience, Aarhus University, Aarhus, Denmark; 3 Department of Physics and Astronomy, Aarhus University, Aarhus, Denmark; 4 Centre for Cognition and Decision making, HSE University, Moscow, Russia; Newcastle University Medical School, UNITED KINGDOM

## Abstract

Despite the clear importance of language in our life, our vital ability to quickly and effectively learn new words and meanings is neurobiologically poorly understood. Conventional knowledge maintains that language learning—especially in adulthood—is slow and laborious. Furthermore, its structural basis remains unclear. Even though behavioural manifestations of learning are evident near instantly, previous neuroimaging work across a range of semantic categories has largely studied neural changes associated with months or years of practice. Here, we address rapid neuroanatomical plasticity accompanying new lexicon acquisition, specifically focussing on the learning of action-related language, which has been linked to the brain’s motor systems. Our results show that it is possible to measure and to externally modulate (using transcranial magnetic stimulation (TMS) of motor cortex) cortical microanatomic reorganisation after mere minutes of new word learning. Learning-induced microstructural changes, as measured by diffusion kurtosis imaging (DKI) and machine learning-based analysis, were evident in prefrontal, temporal, and parietal neocortical sites, likely reflecting integrative lexico-semantic processing and formation of new memory circuits immediately during the learning tasks. These results suggest a structural basis for the rapid neocortical word encoding mechanism and reveal the causally interactive relationship of modal and associative brain regions in supporting learning and word acquisition.

## Abstract 2

This combined neuroimaging and brain stimulation study reveals rapid and distributed microstructural plasticity after a single immersive language learning session, demonstrating the causal relevance of the motor cortex in encoding the meaning of novel action words.

## Introduction

A proposition, which only quarter a century ago would have surprised most neuroscientists, is now supported by an overwhelming amount of evidence: Even the adult human brain is remarkably dynamic, plastic, and reconfigurable. A number of studies have shown how functional and structural alterations at different time scales can be brought about by aspects of the environment, experience, and the overall exercise of human cognitive faculties. Arguably the most remarkable of these faculties is our ability to learn and use language. While much scarcer than data on neural plasticity due to motor learning [1–3], sports [4,5], or spatial expertise [6,7], a few studies have shown that language experience spanning weeks [8,9], months [10], and years [11] produces measurable structural changes in the brain. In contrast, however, we know surprisingly little about the neural basis of rapid, online aspects of learning, despite the fact that children and adults can acquire and start using words almost instantly, after even a single exposure [12]—a feat that is quite unlike the intensive and prolonged practice required by many other skills (including motor ones, e.g., juggling [13,14]).

While both behavioural and neural dynamics [12] suggest functional reorganisation that supports cortical word learning on a minute-by-minute scale, evidence of similarly rapid structural changes is scarce in humans. Although microstructural plasticity has been demonstrated in animals after mere minutes of skill learning [15,16], language acquisition by definition has no suitable animal models and has to be addressed noninvasively in humans. Moreover, we do not know the spatial layout of any such rapid microplasticity. For example, while normal language processing relies on a distributed network of temporal, frontal, and parietal regions [17], existing work on rapid language learning has often failed to capture this—showing instead highly constrained plastic changes in areas that are typically not considered part of the core linguistic network (for example, in primary visual cortex [18]). However, a recent study has shown promising evidence that early cortical reorganisation can be measured after only 6 short word learning sessions, with changes in inferior frontal, middle temporal, and inferior parietal brain regions [19]. Nevertheless, we still lack microstructural evidence to illuminate one of the most central theoretical disputes in language neuroscience—the role of semantic hubs such as the anterior temporal lobe (ATL) or angular gyrus (AG) in language learning and processing, and their relation to modality-specific brain areas, such as the motor cortex [20–24]. A potential reason for this could be the limited sensitivity of common neuroimaging methods to microscale neural changes. While it is unlikely that macroscopic grey matter (GM) modifications take place in such a short time span, animal research [15,16] suggests microscopic modifications of neural tissue, which are difficult to pick up by conventional structural magnetic resonance imaging (sMRI). Diffusion tensor imaging (DTI) is frequently used to assess connectivity but has reduced sensitivity to non-gaussian water diffusion in tissues, which somewhat limits its usefulness in describing early local cortical reorganisation. For example, fractional anisotropy (FA) is a common DTI measure that can be used to infer patterns linked to dendritic and synaptic proliferation, as well as neuronal differentiation in the cerebral cortical tissue during the fetal-neonatal stage of development; at later stages, however, adult cerebral cortical FA is as low as noise level and is not sensitive to cortical microstructure [25]. However, new protocols based on diffusion kurtosis imaging (DKI) [26] possess increased sensitivity to tissue microstructure due to their ability to also capture information about non-gaussian diffusion throughout the brain [27,28]. Non-gaussian diffusion is thought to be a consequence of water movement restrictions caused by cell membranes, organelles, and axonal sheaths [26]. DKI was recently used to link increases in mean kurtosis (MK) and decreases in mean diffusivity (MD) to greater microstructural tissue complexity, and these measures have been shown to be sensitive to GM and white matter (WM) features such as cell shape, size, density, packing, intra and extra-axonal diffusion, tortuosity, and cytoarchitecture [26–33]. For example, immunohistochemistry analyses in animal models have shown MK to index fast proliferation of astrocytes and other glial cells [34,35], which are critical participants in almost all aspects of brain development and function [36], ensuring, among others, neuronal energy supply, insulation, and neurotransmission. A recent study has also shown that MK tracks microstructural differences in the cerebral cortical mantle in the form of neurofilament density (ND) measured using histological image staining [25]. DKI metrics have also been linked to cognitive traits, such as working memory and executive function [37]. Thus, complementing existing MD measurements with MK promises increased sensitivity for detecting the full range of microstructural processes in the brain. Here, we have applied DKI to investigate microstructural plasticity during online language learning, as well as how this reorganisation could be causally manipulated using transcranial magnetic stimulation (TMS).

**Fig 1:**
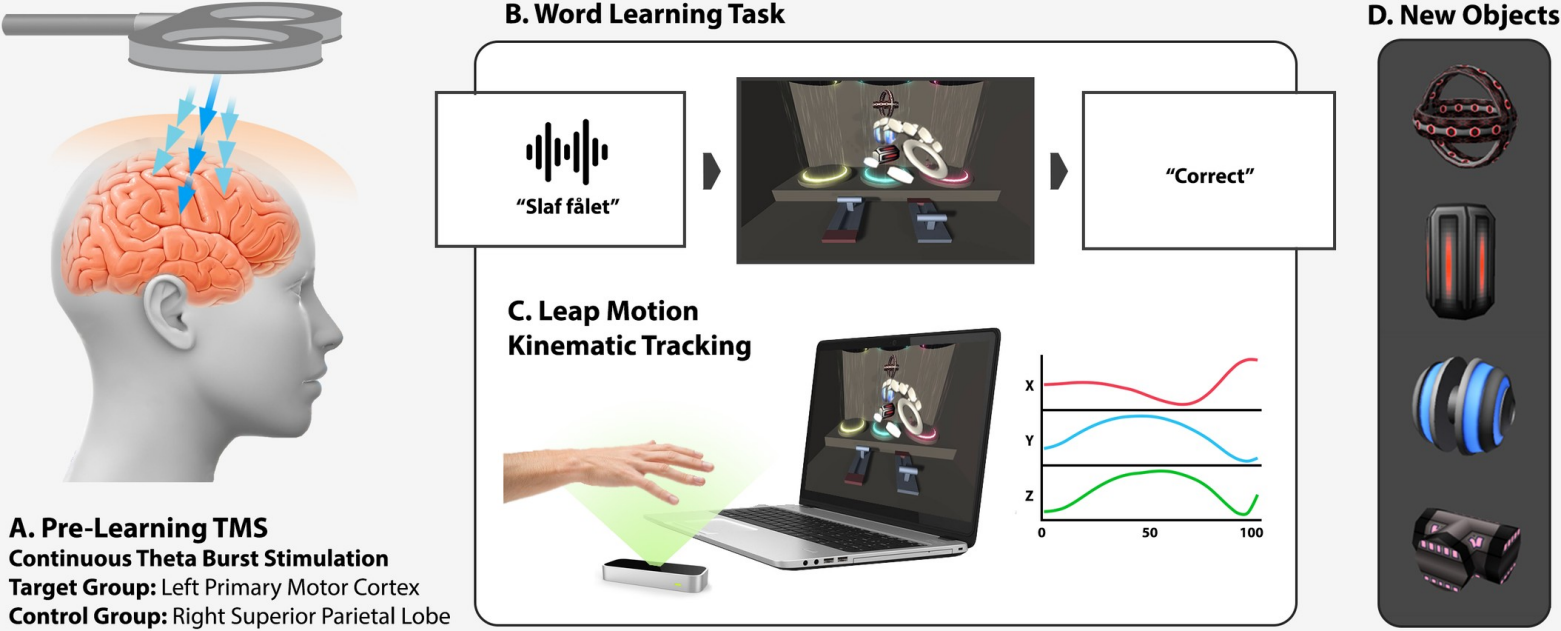
Task design. (A). Two groups of participants underwent 40 seconds of cTBS over either left M1 (primary motor cortex target) or a control site (right superior parietal lobe, subarea 5l) prior to the language learning task. (B). Participants learned new verbs and nouns by playing a 3D computer game in which they used their right hand to interact with objects in the virtual environment. (C). Their learning performance and hand movement kinematics were tracked and quantified throughout using a Leap Motion controller. (D). Illustration of the 4 novel 3D objects. All participants received DKI brain scans directly before and after the language task, as well as after 24 hours, in order to assess learning-induced microstructural remodelling as reflected by diffusion kurtosis changes. cTBS, continuous theta-burst stimulation; DKI, diffusion kurtosis imaging; TMS, transcranial magnetic stimulation.

In this combined DKI-TMS study, we were able to record (as well as externally modulate) rapid microstructural changes occurring within only 40 minutes of language learning. This finding was made possible by innovations in (a) our training protocol, which employed a naturalistic and immersive virtual learning environment (VE), and (b) the application of advanced MR measurement techniques based on DKI, which provide more sensitive biomarkers of structural change than classic techniques such as DTI or T1/T2-weighted sMRI. Forty-seven adult participants (25 male, mean age 22.9 years) were randomly assigned to 2 groups and learned a carefully designed micro-vocabulary consisting of novel action verbs and novel object nouns. They did so implicitly and inductively by playing a 3D game that used optical kinematic tracking to allow participants to interact with and move virtual objects with their hand. (see Fig 1) This active fast-mapping scenario mimicked naturalistic word acquisition much more closely than is possible in other psycholinguistic paradigms that use definitions or simple word-picture association. Learning of action-related language has been linked to intact motor cortex processing [38–46]. Therefore, in order to determine the motor cortex’ role on learning efficacy as well as ensuing neural plasticity, in one group of learners, the motor cortex was left undisturbed (active TMS control area was stimulated—the 5l subarea of the right superior parietal lobe), whereas the other group learned after the left primary motor cortex function was disrupted through TMS stimulation.

By leveraging the above training protocol in conjunction with DKI and state-of-the-art machine learning tools for neuroimaging data analysis, we were able to show that the brain is even quicker than previously thought at adapting structurally to learning new words. Our results reveal structural changes reflected in mean tissue diffusivity and kurtosis (1) in the left ATL and AG within minutes after learning, and (2) in the hippocampal and caudate regions of interest (ROIs) 24 hours after learning. Moreover, by using targeted brain stimulation, we show that plasticity in high-level lexico-semantic hubs (ATL and AG) is linked to unimpaired initial processing in basic modal structures, such as the motor cortex in the present case of action word learning. A complementary whole-brain machine learning analysis of multivariate patterns in GM microstructure confirmed the widespread pattern of learning-induced changes. GM voxels significantly predictive of learning groups (86% mean cross-validation accuracy) included those in motor and supplementary motor areas, bilateral ATL and middle temporal gyrus (MTG), left parahippocampus, left caudate, anterior cingulate cortex, and the right cerebellum. These data are consistent with the view that the ATL and AG “compress” modal semantic information from sensorimotor cortices and serve as pointers to distributed semantic representations, rather than centrally representing or redescribing said semantic information. As such, our results highlight a possible structural neocortical basis for a learning mechanism involving rapid integration of new conceptual information into existing knowledge structures, as proposed by fast mapping theories [47–49].

## Results

Participants learned new object nouns and action verbs by playing a 3D computer game for 40 minutes. Using optical tracking of hand and finger movements, they interacted with a game environment by moving their right hand to pick up virtual 3D objects (4 new objects and, thus, object nouns) and perform novel multicomponential actions on them (denoted by 4 new verbs). For each participant, we recorded DKI images before and after learning (immediately as well as 24 hours later). To assess whether rapid brain plasticity can be causally modulated using TMS, we compared behavioural and microstructural learning outcomes when left M1 was or was not disrupted prior to learning, given this area’s key role in processing action language [38,50,51]. We also recorded behavioural online measures of learning accuracy and hand movement kinematics in the form of complexity and fluidity. Kinematic complexity quantified how complex the hand trajectories were, based on the variance captured by principal component decomposition of participants movements, whereas kinematic fluidity measured how fluid/smooth their movements were (for details, see Methods).

**Fig 2:**
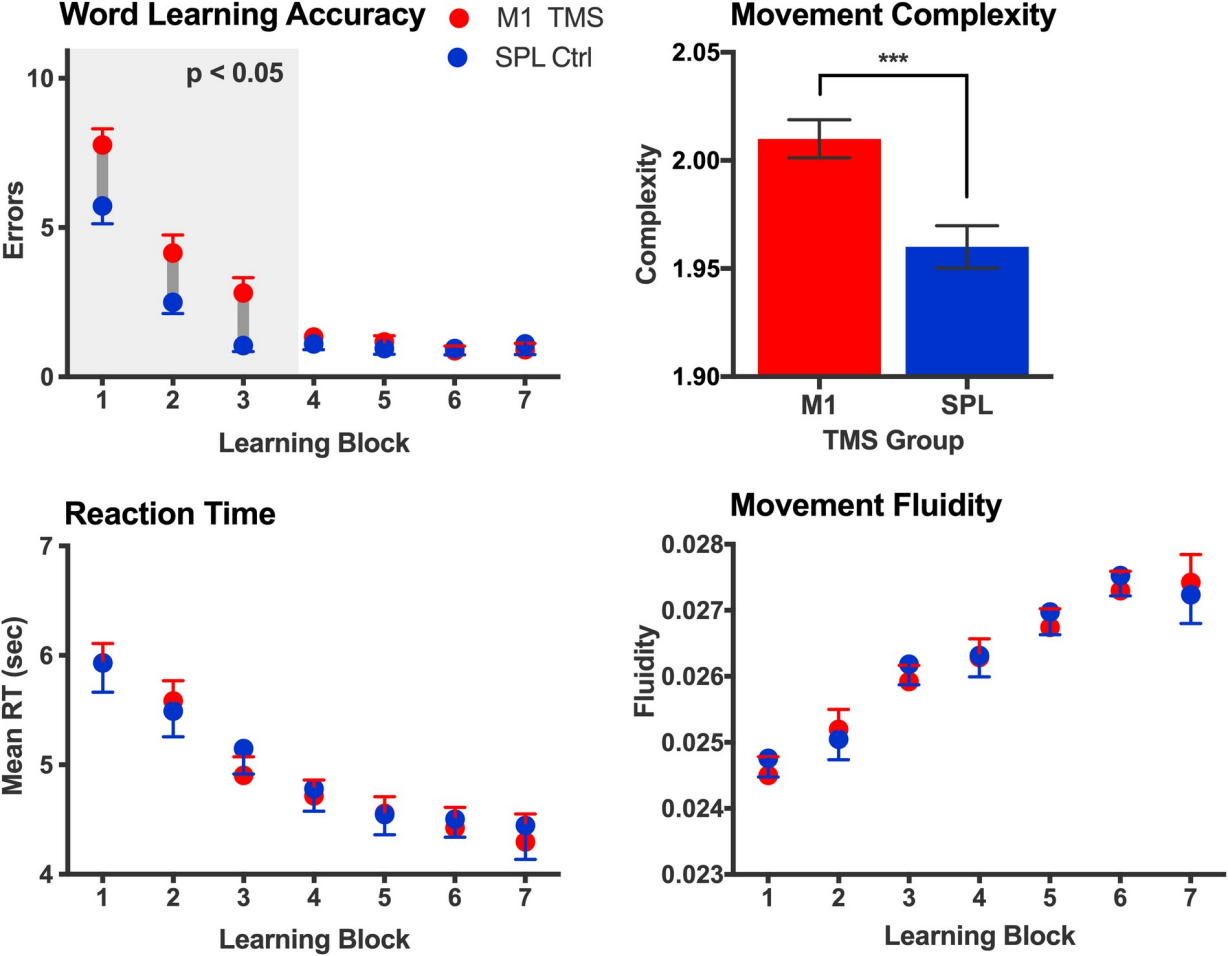
Language learning performance and kinematic measures. Participants who received disruptive TMS stimulation over M1 made more errors during word learning compared to the control group. Moreover, kinematic complexity was greater in these participants compared to controls, indicating more complex hand trajectories and likely greater cognitive uncertainty. At the same time, both groups had comparable response speed as well as movement fluidity/smoothness, indicating that TMS affected cognitive performance, as opposed to just low-level motor processes. Error bars represent SEM. The data underlying this figure are provided on the Open Science Framework (https://osf.io/k9ngw). RT, reaction times; SEM, standard error of the mean; TMS, transcranial magnetic stimulation.

After false discovery rate (FDR) correction for multiple comparisons, behavioural results show that learning performance/accuracy improved over time in all participants [F(1,6) = 108.68; *p* < 0.0001], as did their mean reaction times (RT) [F(1,6) = 61.07, *p* < 0.0001], and hand movement fluidity [F(1,6) = 30.78, *p* < 0.001]. However, whereas no group differences emerged in terms of mean RT (all corrected *p* > 0.05), accuracy was worse in those participants who received disruptive M1 TMS stimulation, compared to controls [F(1,2) = 16.61; *p* < 0.001]. Moreover, while all participants performed equally fast and fluid hand movements, the M1-stimulated group exhibited path trajectories of greater complexity compared to controls [F (1,2) = 36.31; *p* < 0.0001]. Thus, while TMS stimulation did not affect participants’ gross speed or low-level motor kinematics/fluidity, it caused worse accuracy and more complex hand trajectories, likely reflecting greater cognitive uncertainty (see Fig 2). Indeed, these findings are consistent with earlier TMS research using the continuous theta-burst stimulation (cTBS) protocol, which confirmed that while this M1 stimulation impacts cognitive processes, motor-evoked potentials, and functional brain activity, it does not disrupt execution of manual movements as such [52].

**Fig 3:**
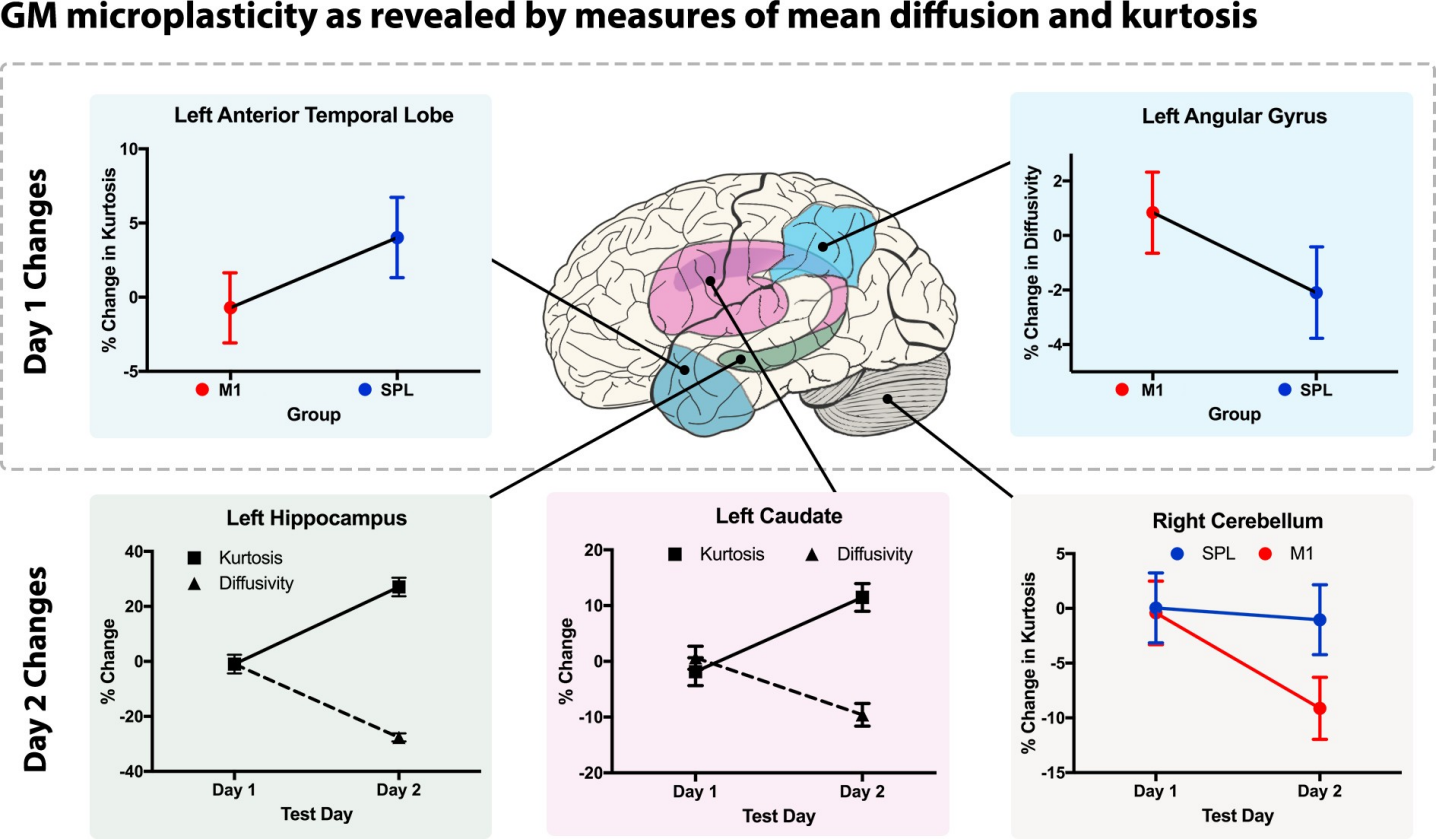
Results of ROI analysis. Statistically significant GM microplasticity as revealed by measures of mean diffusion and kurtosis changes (relative to baseline scan) evident already on the first follow-up scan (Day 1): In the control group, whose motoric learning was not impaired, there was an overall mean kurtosis increase in the left ATL and a diffusivity decrease in the left AG ROI (i.e., main effect of Group). Moreover, 24 hours after language learning (follow-up scan on Day 2), mean diffusivity and kurtosis differences were evident in the left caudate and left hippocampus in both learning groups (main effect of Test Day/Session). Finally, in participants who received disruptive M1 TMS, there was an overnight (after 24 hours) decrease in mean kurtosis in the right cerebellum, likely reflecting more effortful learning of action semantics through the cerebellar error-based encoding mechanism (Group × Day interaction). The above GM differences are relative to the pre-learning baseline measurements (i.e., they express percent change from the baseline). Error bars represent 95% confidence intervals. The data underlying this figure are provided on the Open Science Framework (https://osf.io/k9ngw). AG, angular gyrus; ATL, anterior-temporal lobe; GM, grey matter; ROI, region of interest; TMS, transcranial magnetic stimulation.

Based on past research [17,21,53–55], we looked for microstructural changes in a left-lateralised language network comprising ROIs in inferior frontal gyrus (IFG), Wernicke’s area, the AG, and ATL. We also investigated 4 further learning-relevant ROIs: the hippocampal area, the caudate, and—in the domain of action semantics, specifically—the primary motor cortex and cerebellum. A 2 × 2 (Group × Test Day) ANOVA analysis of these 8 a priori defined GM ROIs revealed significant MD and MK differences (percent change values measured relative to pre-learning baseline; all values are FDR corrected). Namely, we observed a significant main effect of Group in the left ATL [F(1,2) = 7.52, *p* = 0.022] and the AG [F(1,2) = 7.46, *p* = 0.023], which were due to the fact that participants from the control group (whose learning was not impaired by M1 stimulation) displayed a significant MK increase in the ATL and an MD decrease in the AG. The absence of a significant main effect of Test Day (*p* = 0.37) in these ROIs indicates that the above differences were evident already on day 1 and remained during the subsequent day 2 scan. Next, we observed significant main effects of Test Day in the left hippocampal ROI [MD: F(1,2) = 657, *p* < 0.0001; MK: F(1,2) = 140, *p* < 0.0001] and the left caudate [MD: F(1,2) = 49, *p* < 0.0001; MK: F(1,2) = 57, *p* < 0.0001]. This effect was due to the fact that both test groups showed a significant MK increase and an MD decrease in these 2 ROIs on day 2 (24 hours after learning), whereas no differences were visible on day 1. Finally, in the Right Cerebellum ROI, we recorded a significant main effect of Group [MK: F(1,2) = 8.46, *p* < 0.007] and Test Day [MK: F(1,2) = 11.15, *p* < 0.004], which were further qualified by a significant interaction between Group and Test Day factors [F(1,4) = 6.76, *p* = 0.01]. This was because participants who received left M1 TMS (but not controls) exhibited a decrease of tissue MK in the right cerebellum 24 hours post-learning but not on day 1. These findings are illustrated in Fig 3. Inspired by a helpful comment from an anonymous reviewer, in addition to these a priori ROI analyses, we conducted an additional post hoc analysis for a spherical ROI (r = 10 mm) centred over the coordinates of our right SPL control region. No significant main effect or interaction emerged for this ROI (all *p* > 0.05), indicating that there was no general (i.e., nonspecific) effect of TMS stimulation on diffusion or kurtosis measurements. In other words, TMS on its own did not cause any measurable microstructural reorganisation over the active control site, supporting the view that the above reported effects are indeed a consequence of the linguistic/semantic learning procedure.

**Fig 4:**
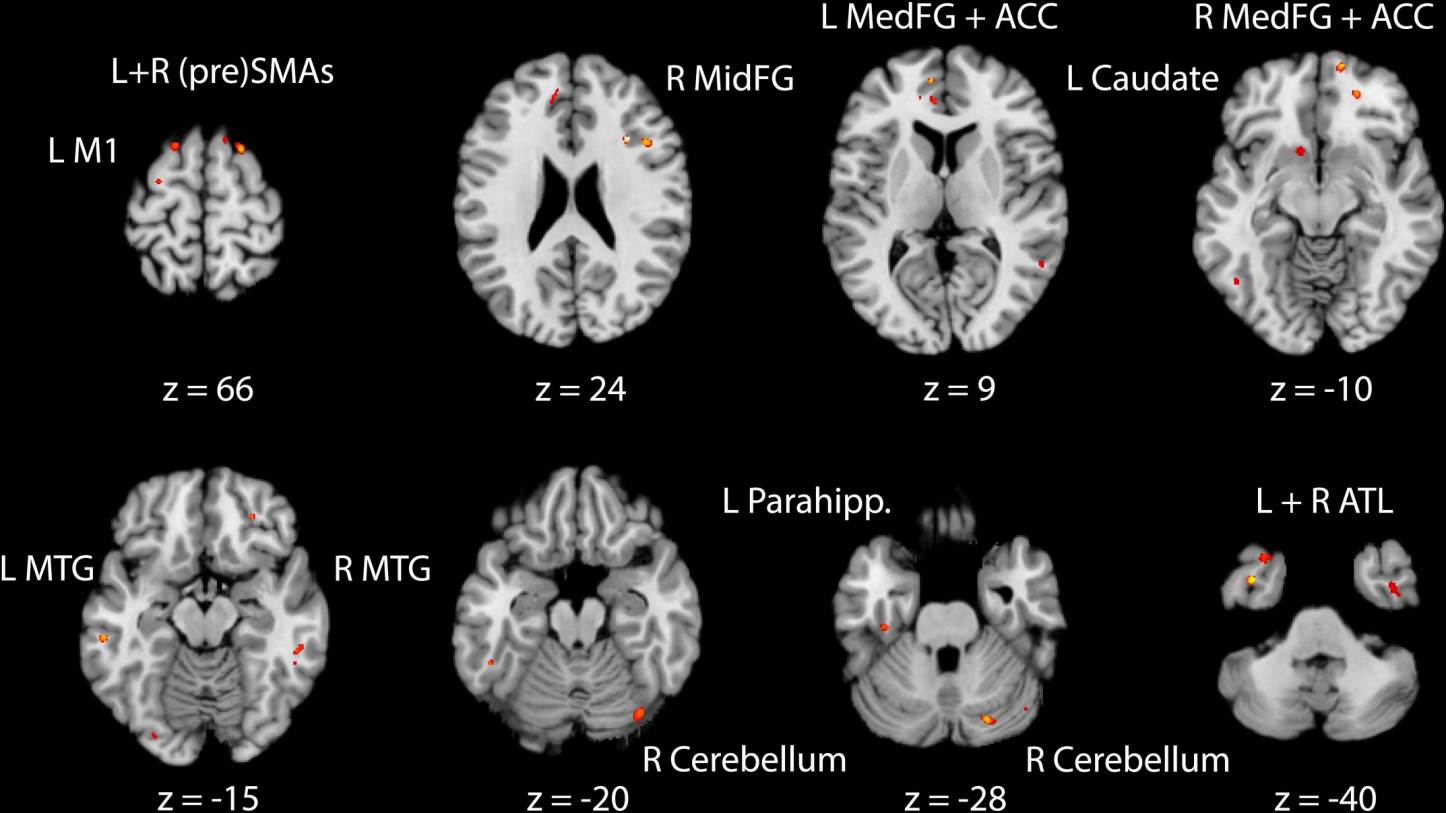
MVPA analysis of learning-induced GM changes (relative to the pre-learning baseline). GM voxels, which were significantly predictive (86 +− 1.5% mean accuracy) of the 2 learning groups (M1 and SPL) already on day 1, were found in bilateral MTG, bilateral ATL, anterior cingulate, medial PFC, bilateral (pre)SMAs, left M1, left caudate, left parahippocampus, and right cerebellum. The data underlying this figure are provided on the Open Science Framework (https://osf.io/k9ngw). ACC, anterior cingulate cortex; ATL, anterior-temporal lobe; FG, frontal gyrus; GM, grey matter; MTG, middle temporal gyrus; MVPA, multivoxel pattern analysis; PFC, prefrontal cortex; SMAs, supplementary motor areas.

We next conducted a whole-brain analysis using machine learning classification of the microstructural changes, without any a priori selection of cortical GM. Learning-induced DKI change values (between the pre-learning and day 1 post-learning scan) were fed into a SpaceNetClassifier [56], implementing combined TV-L1 priors, which yield both structured and sparse regression coefficients [57,58]. We were able to classify the 2 language learning groups based on the patterns of kurtosis and diffusivity changes with a very high accuracy of 86% (SD = 1.5%). Brain voxels whose features were significantly predictive of the learning group membership were found in left M1, right cerebellum, and bilateral (pre) supplementary motor areas (SMAs), consistent with the literature on action language processing and motor learning. In addition, we saw predictive voxel clusters in left parahippocampus and bilateral MTG, anterior cingulate, and medial prefrontal cortex (PFC). Finally, multivoxel pattern analysis (MVPA) also highlighted the left caudate, as well as the bilateral ATL, as regions in which microstructural plasticity was predictive of learning group (see Fig 4).

Thus, combined results from behavioural, ROI, and MVPA analyses show that a single 40-minute session of immersed language learning brings about measurable plastic changes throughout the lexico-semantic brain network. This rapid plasticity is predictive of different groups’ variable accuracy and learning performance and could be externally modulated using noninvasive brain stimulation.

## Discussion

Despite its central importance in our lives, our ability to quickly and efficiently learn words and their meanings remains poorly understood. While we know that children and adults can learn words within mere minutes via the so-called fast-mapping mechanism [59,60], most existing studies of structural brain reorganisation describe plasticity associated with weeks, months, and years of language use ([10,11,55,61]—however, note [19], where more constrained cortical changes were observed after an hour of flashcard-based word learning). In this study, we sought to characterise (and externally modulate) the cortical profile of the earliest structural signatures of word learning. We show evidence of rapid microstructural plasticity throughout the brain’s lexico-semantic network, occurring within less than an hour of immersed language learning. Semantic processing is word category dependent—e.g., words referring to visual percepts will be partly subserved by the visual cortex, whereas aspects of words describing sounds will be represented by the auditory cortex [20,21,62], etc. Because of this, it is important to control for the semantic category of lexical items—in this study, we focused on the domain of action-related language as a case study of word learning. Because previous research has shown that processing action-related sentences relies on the motor cortex [38,51,63–66], we introduced a controlled modulation of learning using the TMS method. Therefore, we could test the causal consequences of referentially grounded (i.e., motoric) versus nonmotoric learning on resulting brain representations and plasticity by applying disruptive TMS stimulation to the primary motor cortex (left M1). Our prediction was that magnetic stimulation of this key action-semantic cortical node will disrupt the acquisition of new action language and formation of related semantic representations. We found that not only did this intervention interfere with behavioural indices of action word learning in the M1-stimulated participants but it also prevented microstructural changes in semantic brain areas such as the left ATL and AG. Finally, we used machine classification to show that multivariate plasticity patterns reliably predict learning group membership in areas associated with language processing, memory formation, and executive control. Our results not only speak to the promise of using DKI as a sensitive tool to measure microscopic structural alterations (complementing standard diffusion imaging protocols) but also show how these methods enable us to better elucidate the rapid temporal dynamics of human cortical plasticity, more generally, and the acquisition of new lexical semantics, specifically. Below, we discuss these findings in more detail.

### Localisation of plastic changes

#### Temporal and inferior parietal areas

The left ATL and AG are key regions thought to enable the processing of familiar and novel semantics, such as word meaning and conceptual representations [20,21,50,61,63,64]. A wealth of brain research supports the idea of widely distributed representations of knowledge, with different modal areas encoding specific conceptual features [22,67,68]. At the same time, most current theories argue for the necessity of semantic “hubs” or “convergence” regions, which bind together discrete property regions (such as perceptual and motor ones) into such broadly distributed circuits [20,21]. The ATL and the AG are widely considered as prime candidates to implement such a semantic convergence/hub role. Evidence supporting this suggestion comes, for example, from patients with left ATL damage who fail to acquire new semantic associations [47], from semantic dementia [22,69], as well as from numerous word reading and comprehension studies (for reviews, see [20,21]). The results of our investigation are in line with the hypothesis that the left ATL and AG support rapid neocortical encoding of lexical semantics through a fast-mapping mechanism: Following word learning, we saw significant plastic changes (reflected by both MK increase and MD decrease) in both of these ROIs.

A question, however, which ties into a key theoretical debate, is whether association areas such as the ATL and AG (1) compress modal information from sensorimotor cortices (acting as “pointers” to this distributed information) [70–72], or (2) themselves represent conceptual semantic information [73,74]. Our data show that plasticity in left ATL and AG ROIs was evident only in individuals who could learn action language motorically, whereas no significant changes from baseline emerged if participants’ M1 was disrupted prior to learning. Thus, it appears that plasticity in these semantic hubs was dependent (at least in the present case of action-word learning) on unimpaired processing in modality-specific (here, motor) regions. These data may suggest that the ATL and AG act to provide a link between a word form and distributed modal information, rather than redescribe such information into self-standing abstract semantic representations. In other words, if memory encoding is disrupted in motor cortex by TMS [75–78], less modal information is available that can be integrated in the association cortices. Machine classification based on multivariate patterns in diffusion kurtosis data additionally demonstrated that voxels in bilateral ATL and MTG could be used to predict whether an individual learned novel words with or without prior TMS disruption of M1 (which led to decreased online learning accuracy). Meta-analytic neuroimaging studies of semantic processing, such as in Binder and colleagues (2009), identified the MTG (in addition to AG, PFC, parahippocampal, and retrosplenial cortex) as a key semantic site—especially by virtue of receiving extensive (supra)modal cortical input, thus supporting a likely role in multimodal integrative processes. Moreover, given its proximity to visual motion processing areas, the MTG has been shown to store action-related semantic knowledge about visual properties of movements, as well as manipulable objects and tools [79–84]. This visuo-motor integration function of the MTG might be particularly relevant in the context of the present study, in which participants had to learn word forms and meanings relating novel objects to nouns and action-sequences to verbs.

#### Prefrontal and hippocampal areas

ROI analysis of mean left hippocampal plasticity showed significant learning-induced reorganisation between first- and second-day scans. Moreover, the MVPA analysis indicated that multivariate patterns of plastic changes within the left parahippocampus were reliably predictive of participant groups associated with differing levels of learning accuracy and performance. In addition to changes in hippocampal brain structures, we also observed diffusion kurtosis differences in medial PFC (together with the anterior cingulate cortex (ACC)) bilaterally. This prefrontal microplasticity likely reflects memory mechanisms that rely on attentional and executive resources which, in tandem with medial temporal structures, underlie successful semantic encoding [85–87]. Given that our novel word labels and meanings were not only similar to each other but also mapped onto existing morphosyntactic, phonological, and orthographic patterns of the participants’ native language, the PFC together with hippocampal structures may contribute to successful concept interference resolution (pattern separation) and associating novel information with existing lexico-semantic knowledge. Indeed, the available literature points to an intimate link of the medial PFC with (para)hippocampal and retrosplenial declarative memory structures [88], and a role in forming short-term associations (over seconds and minutes, in animals [89,90]), retrieval of episodic knowledge [91], and memory monitoring [92].

The left caudate is another structure that underwent measurable microstructural reorganisation after language learning. While long associated with motor processes and inhibitory control of action [93,94], the caudate also plays an important role in nonmotor functions including procedural and associative learning [95,96], as well as language [97–99]. Especially in the left hemisphere, it is prominently associated in neuroimaging studies with bilingual language processing, lexical semantic control [100–102], and verbal learning capacity [103]. Based on this past research and on our finding of rapid plasticity in the left caudate (in both univariate and multivariate analyses), a plausible role of this cognitive control area could be in selection of competing lexical and phonological forms both between similar novel, as well as between novel and existing verbal entries (which all conformed to the same orthophonological rules of the participants’ native language). Supporting this notion, past studies in L2 learners and bilinguals indicate greater GM density and connectivity indices in the caudate, which correlate with better phonemic fluency [61,99,104,105]. Interestingly, abnormalities and asymmetries of GM volume in the caudate nucleus have been linked to specific language impairment and developmental language disorder and are negatively correlated with nonword repetition skills [106–110].

#### Motor brain areas

Based on our own [38–42] and other labs’ studies [43–46] of normal language processing, we expected that learning novel action language (especially in the context of immersive training) would result in lexico-semantic memory traces, which are partly encoded by the brain’s motor structures. Consequently, we expected to see differential motor encoding between the M1 TMS group, whose M1 was disrupted during learning, and the active controls who could learn unimpaired. In support of this prediction, we demonstrate that plasticity in the left M1, bilateral (pre)SMAs, and the right cerebellum can be used to accurately classify participants’ membership in the 2 TMS groups soon after learning. Additionally, ROI analyses indicated that 24 hours after encoding MK values of the right cerebellum decreased significantly in the M1 TMS group but not in controls.

Functionally, the SMA has been shown to store and process information needed for planning, imagining, and performing more complex, multistep action sequences [111–114]. Moreover, during planning and motor imagery tasks, SMA shows a suppressive influence on M1, likely preventing the overt generation of movements [63,115,116]. In the language domain, it has been shown that action word processing is associated with and can be decoded from SMA activity [117] and that verb reading triggers increased effective connectivity between left M1 and bilateral SMAs [63]. With regard to the cerebellum, research indicates its integral role in motor planning and adaptation through subserving error-based learning, specifically, and more generally in the learning of procedural sequences [118]. With respect to language, its role is well established during planning of speech, mental rehearsal, and lexico-semantic processing of action-related language (e.g., in genetic atrophy of cerebellum [119]). Thus, the cerebellum seems to underpin trial-by-trial learning based on, among others, motor error signals due to wrong internal prediction models [120,121], unpredicted sensory feedback [122,123], or adaptation to novel visuo-motor transformations [124]. It is interesting to note that in our study, we observed increased cerebellar plasticity specifically for the group that exhibited more errors, and more complex hand trajectories, when learning novel action verbs and motor sequences (i.e., the M1 TMS group). Moreover, our learning task involved right hand movements, and plasticity was evident (in both the ROI-based and MVPA analyses) specifically in the right cerebellum, which selectively processes ipsilateral motor information. Plausibly, the rapid cerebellar plasticity observed in the M1 group is reflective of an error-based encoding mechanism associated with the mismatch between sensorimotor predictions launched in response to novel action verbs, the diverging sensory feedback, and subsequent error correction.

### DKI as a measure of structural plasticity

DKI is a leading tool for noninvasive imaging of tissue microstructure [26,125], and its microstructural sensitivity is documented in numerous animal studies linking DKI metrics to tissue microarchitecture, as supported by microscopy-based histological analysis [126–128]. Kurtosis reflects a complex combination of a number of tissue properties on the cellular scale, including neurite density and dispersion, as well as intracellular and extracellular diffusivities (see, e.g., [129,130]). The intracellular diffusivity, in turn, is affected by axonal varicosities, dendritic spines, and undulation, while the extracellular diffusivity depends on axonal radii and packing, among others. Short-time scale (minutes or less) microstructural remodelling in response to stimuli has been demonstrated by direct observation in cell cultures [131] and in brain slices [132]—these changes include increased tissue compartmentalisation and complexity, leading to a decrease in overall diffusivity and increase in MK, precisely as observed in this study. DKI measures—both MK and MD—have been suggested to reflect GM and WM features at both tissue and cytoarchitecture level, such as cell shape, size, density, within-cell and across-membrane diffusion, etc. [26–33]. Similarly, DKI metrics track ND obtained using histological staining across multiple distinct cortical regions [25]. Animal studies have shown it to reflect proliferation of neuroglia, most importantly astrocytes [34,35]. Although it cannot be confirmed at the current stage, it might be the rapid migration of astrocytes that underpin fast plastic changes required by learning [36], since they play a critical role in supplying neurons with oxygen, in axonal insulation, and in neurotransmission [133], all key factors involved in rapid functional reorganisation of neuronal circuits for the creation of new representations through modifying local connectivity. On a more cautious note, as a noninvasive tool, DKI provides indirect indices of tissue microstructure, which limits our interpretation of DKI-based findings in terms of exact tissue cytoarchitecture [129]; this remains a target for future studies that will have to use animal experiments combining DKI with direct cytological measures at a microscopic level. Nevertheless, the DKI technique appears to be a powerful tool for the study of human brain plasticity when combined with strong experimental design, and hypothesis and interpretation, as backed by a growing number of ex vivo validation studies and shown by the present data.

## Conclusions

Understanding the dynamics of neural plasticity and its role in learning is a fundamental question in neuroscience and lies at the core of how neural structure and function interact. Characterising the earliest signatures of such brain reorganisation is particularly important in the language domain, given that both children and adults can learn new words and word meanings with remarkable speed and ease, whereas any deficits in this poorly understood ability have grave consequences for the individuals affected and for society. To address this issue, our study made use of a novel VE word learning task in conjunction with DKI, which, compared to classic sMRI approaches, has increased sensitivity to brain tissue microstructure [26–28]. We found significant cortical plasticity across multiple brain systems occurring after only 40 minutes of word learning; moreover, we show how this plasticity can be modulated using noninvasive brain stimulation. Crucially, we were able to capture widespread diffusion kurtosis changes, indicative of microstructural modifications in GM tissues, in prefrontal, temporal, and parietal cortical nodes, which are known to subserve integrative semantic processing and control across a range of linguistic and learning tasks. Our results thus provide a possible structural basis for the rapid neocortical encoding mechanism proposed by fast-mapping theories [23,47,49,60] and reveal the causally interactive relationship of modal (e.g., M1) and associative (e.g., the ATL and AG) brain regions in supporting the initial stages of lexical and conceptual learning. While many unanswered questions remain to be tackled by future studies, such as how long lasting these alterations are [134–136] and what their exact biological basis is (for a review, see [16]), the present investigation opens a new window into the adaptive nature of our linguistic brain, demonstrating reorganisation that is much faster and more distributed than previously thought.

## Methods

### Participants

Using the G-Power statistical software [137], assuming a small to medium effect size observed in prior related work [19,38,138,139] and a significance threshold of 0.05, we calculated a minimum sample size of 20 participants per group. Allowing for possible sample attrition, we recruited a total of 47 adult Danish participants through the institutional SONA Participant database (www.sona-systems.com). All of them were right-handed, had normal vision, and were neurologically healthy, with no history of language disorders. These were randomly assigned to 2 groups through SONA: 26 participants learned words after disruptive M1 TMS stimulation (13 males; age = 23.5 ± 2.84), and 21 participants after control area (SPL) stimulation (12 males; age = 22.3 ± 2.24). All study protocols were approved by the Central Denmark Research Ethics Committee (biomedical research ethics approval No. 1-10-72-95-16) and were conducted in full accordance with the principles expressed in the Declaration of Helsinki. Participants were paid for their time and signed an informed consent form.

### Experimental procedure overview

Each participant underwent the same multicomponent testing procedure. First, a T1 anatomical image was acquired to facilitate TMS neuronavigation. Then, they would move to the TMS lab where the cTBS protocol was administered. Following this, they moved to the behavioural testing room where they played the language learning game for around 40 minutes. The microstructural MR scanning was performed immediately after this. Thanks to all 3 labs (TMS, MRI, and behavioural) being adjacent to each other, the gap between each step was minimal: 5 minutes between TMS and learning, and under 10 minutes between the learning task and MRI acquisition.

### Stimuli and learning task

Participants learned new words by playing a 3D VE game. Rather than learning new words artificially through definition, as is common in classrooms and linguistic experiments, our participants learned the way a child naturally does—by associating word labels with actions they perform or with objects they see. For this, we used a computer-based learning setup, which incorporated a small camera that recorded participants’ right-hand movements, transposed them into a virtual 3D space, and thus allowed interaction with virtual objects in the game. By using this active paradigm, participants learned the meaning of 4 new action verbs (“plit,” “slaf,”” klur,” and “fryp”) and 4 new object nouns (“triffen,” “balsen,” “fålet,” and “skrullet”). All of the novel words conformed to the rules of the participants’ native language (i.e., Danish phonology, orthography, and morphosyntax)—because of this, our Danish participants could, based on pseudoword forms alone, identify their grammatical category (verb versus noun) but not their meaning. The novel nouns referred to unique 3D in-game objects (Fig 1D), which could be manipulated using novel action sequences denoted by the new verbs. Each of these verbs denoted a 3-step action consisting of selecting and taking the correct object, putting it on 1 of 2 designated platforms, and then pushing 1 of 2 virtual levers (importantly, the motor routines were novel and did not correspond to any existing native verb) (Fig 1B). The learning task mimicked naturalistic language learning in that it was (a) active, with associations discovered on the fly through induction and trial by trial feedback, (b) contextual, such that new information was learned inferentially by comparison to previous information (“if not A, then B”), and (c) constructive, with information being similar yet sufficiently unique from previous knowledge (e.g., novel verbs were not simply new labels for existing lexicalised actions).

Throughout the learning session, online kinematic and movement data were acquired using the Leap Motion controller (Leap Motion, San Francisco, California), which sampled the position of the right palm centroid at a frequency of 100 Hz, and an average tracking precision of 0.2 to 1.2 mm [140,141]. The entire learning task lasted for 40 minutes and included a practice and familiarisation stage and subsequent 13 test blocks of 16 trials each. On each of these trials, a participant would see and hear a novel action sentence following the “Verb the Noun” pattern (e.g., “Slaf triffen”), and then see a visual 3D scene containing 2 platforms and 2 levers and the target object (e.g., “triffen”) among several distractors. By trial-and-error learning, participants would infer the meaning of the new words—if successful, they would see a “correct” feedback message at the end of the trial. Similarly, an “incorrect” feedback message would be displayed for 500 ms as soon had the participants made a mistake (i.e., immediately after picking the wrong object or performing the wrong action with the correct object). Specifically, a trial would be considered an error trial if a participant picked up the wrong object, or placed it on the wrong platform, or activated the wrong lever (Fig 1). In other words, a trial was correct only if the whole action sequence was correct. This immediate delivery of error feedback significantly simplified word learning—i.e., participants could infer whether they misunderstood the meaning of a noun as soon as they grabbed the wrong novel object and, conversely, a hypothesis about the meaning of a to-be-learned verb could be disconfirmed as soon as they performed the wrong action sequence/sequence segment. As expected based on past work showing verbs are more difficult to acquire than nouns [142], our participants could learn the noun-to-object mappings very easily already during the practice block, whereas the more abstract verb-to-action-sequence mappings took much longer. RT were measured from the onset of the trial to the end of the required action sequence (or until an error was made)—we ensured that all participants started each trial with their hand in the same start location (resting the palm on a marked spot on the desk). Thus, all recorded kinematic trajectories had the same origin and the same RT measurement onset (triggered by lifting the hand from the start marker on the desk). Finally, all object and action labels (i.e., verbs and nouns) were rotated in a counterbalanced fashion between subjects to avoid potential confounds due to any idiosyncratic effects of particular word form-meaning pairings.

### Transcranial magnetic stimulation

To causally investigate the susceptibility of early microstructural brain plasticity to modulation by TMS, we delivered cTBS [143]—a protocol that has been shown to decrease neural excitability for around 45 to 50 minutes after the TMS application [143–145]. cTBS was delivered 5 minutes before the learning task, thanks to adjacent TMS and behavioural testing rooms. Given the previously demonstrated causal role of M1 in processing and representing action language, the target participant group had their left-hand M1 area disrupted before learning. The exact M1 hotspot was determined individually as the point most reliably producing MEPs of at least 50 μV in 5 out of 10 trials in the right-hand FDI muscle. The active control group received TMS stimulation over the right SPL (subarea 5l, MNI: 12, -58, 78 [146]), chosen as an easily accessible TMS site with least reported associations to language learning. While finding the perfect cortical control for a language learning task is very challenging due to the extensive cortical activation observed during linguistic processing (see [147]), studies that combined anatomical, resting state, and functional MRI, as well as functional connectivity data indicate that the subregion 5l of the right SPL is a good nonlinguistic control site, given that it primarily subserves processing of visual attentional and motion information (see [148]).

The stimulation was delivered using a MagPro X100 stimulator (MagVenture A/S, Farum, Denmark) and a 97-mm figure-of-eight coil (held at a 45-degree angle from the midline). In particular, 600 biphasic pulses were delivered in bursts of 3 every 200 ms (i.e., 50 Hz), using an intensity equalling 80% of an individual’s resting motor threshold (rMT). The mean stimulation intensity used was 29.6% (±5.4%) of maximum stimulator output. Throughout, individual T1 anatomical scans and an online frameless navigation system (eXimia Navigated Brain Stimulation, Nexstim, Helsinki, Finland) were used to ensure precise coil positioning and stimulation.

### Behavioural analyses

Word learning performance was assessed through RT, accuracy/error rate, and kinematically through movement fluidity and complexity. Kinematic complexity was defined as the entropy of the proportion of variance contained in the first component obtained from the principal component analysis (PCA) of hand positional data. Movement fluidity, in turn, was defined as the ratio of velocity and acceleration of normed and averaged kinematic data and captured the smoothness of hand trajectories. For details of the implementation of these measurements in MATLAB’s MoCapToolbox, please see Burger and Toiviainen [149]. Mean values of above parameters were entered as dependent variables in a mixed random effects model with the fixed effects of Time (learning blocks 1 through 7) and Group (M1 TMS versus Active Control), an interaction between the two, and nested random effect of Subject and Time, to account for individual differences in learning. Results were FDR corrected using the Benjamini–Hochberg method [150].

### Diffusion kurtosis imaging and processing

Immediately after the learning task, participants were moved to the adjacent MRI lab for scanning. Brain scans were performed on a 3T Siemens Tim Trio scanner equipped with a 32-channel head coil, with foam padding used to reduce head motion during acquisition. For each participant, we acquired a single high-resolution T1-weighted anatomical image (1 mm isotropic voxel size) using the MPRAGE sequence (TR/TE = 2,420/3.7 ms), producing 176 slices in the sagittal plane with a matrix size of 256 × 256. We also acquired diffusion kurtosis images 3 times (before, immediately after, and 24 hours after learning) using the following parameters: TR/TE = 4,200/98 ms, voxel size = 2 × 2 × 2 mm. Volumes had 66 axial slices with a matrix size of 96 × 96. Each of the 3 scans included 9 b = 0 images, with the remaining images acquired along 201 diffusion-encoding directions with shells positioned at 5 b-values (15 × b700, 30 × b1000, 21 × b1200, 60 × b1500, 75 × b2500 s/mm2).

DKI data were preprocessed using an in-house MATLAB pipeline that drew upon FSL functions (http://www.fmrib.ox.ac.uk/fsl) and the unring package (http://www.bitbucket.org/reisert/unring) with the following steps: denoising and Rician noise correction [151,152], Gibbs-ringing correction [153], combined motion, Eddy-current, and EPI-distortion correction using the reverse PE b0 images and bias-field correction [154]. The preprocessed multi-shell data were used to generate multi-tissue (WM, GM, and CSF) probability maps. Calculation of DKI parameters was performed in Matlab (The Mathworks) using standard methods for DKI estimation [26] as outlined in, e.g., Hansen and colleagues [29,31].

### Univariate ROI analysis

We analysed MK and MD values extracted from ROIs shown in the literature to be key linguistic processing areas [21,53]. Specifically, we used the SPM 12 toolbox (https://www.fil.ion.ucl.ac.uk/spm) and the Juelich Anatomy Toolbox [155] to get the default ROI masks for left IFG, left caudate, right cerebellum exterior, left (para)hippocampus, and left AG. Additionally, spherical ROIs were created (r = 10 mm) over the following mean MNI coordinates: left hand M1 (-40–25 55), left Wernicke’s area (60–36 18), and left ATL (-50 6–20). To ensure minimal processing of native space DKI metrics, all ROI masks were defined in standard/MNI Atlas space and were then transformed into each individual’s native space, where for each ROI, we extracted MD and MK values. To guard against artefacts due to partial volume effects, GM tissue probability maps were used to exclude voxels, which were less than 90% likely to be in GM. Notably, DKI values vary naturally both between individuals as well as across different cortical areas [25,156,157]. Because we were presently interested in task-induced microplasticity, as opposed to non-task-specific anatomic variation, we calculated for each participant the delta/difference DKI values, expressed as the percent change in either of the post-learning DKI images, relative to the baseline (pre-learning) scan. These percent change values were the input for the ANOVA used to analyse mean ROI values using the factors of Group (M1 TMS versus Control) and Test Day (1 versus 2). FDR correction was applied to control for multiple comparisons when analysing both diffusivity and kurtosis [150].

### Whole-brain multivariate analysis

Machine learning classification of diffusion kurtosis parameter maps was performed without any a priori parcellation of the brain. Each individual’s GM DKI images were transformed into standard MNI space using SPM (http://www.fil.ion.ucl.ac.uk/spm), and then learning-induced changes were calculated for day 1 and day 2 images, relative to the pre-learning baseline scan (i.e., the input images for the analysis contained the delta/difference values between the baseline scan and the respective post-learning scan on day 1 or day 2). Using the Nilearn Python package [158], these DKI difference images were standardised and fed into a SpaceNetClassifier [56,58] with default parameter values, implementing combined TV-l1 priors (with l1 ratio = 0.05), which yield both structured and sparse (i.e., zero in all nonpredictive voxels) regression coefficients. ANOVA univariate feature selection was used, with a screening percentile of 20. MVPA model performance (classification accuracy) was tested using a 10 stratified K-fold cross-validation procedure, as implemented in the Scikit-learn package [159].

## Abbreviations

AG: angular gyrus
ATL: anterior temporal lobe
cTBS: continuous theta-burst stimulation
DKI: diffusion kurtosis imaging
DTI: diffusion tensor imaging
FA: fractional anisotropy
FDR: false discovery rate
GM: grey matter
IFG: inferior frontal gyrus
MD: mean diffusivity
MK: mean kurtosis
MTG: middle temporal gyrus
MVPA: multivoxel pattern analysis
ND: neurofilament density
PCA: principal component analysis
PFC: prefrontal cortex
rMT: resting motor threshold
ROI: region of interest
RT: reaction times
sMRI: structural magnetic resonance imaging
TMS: transcranial magnetic stimulation
VE: virtual environment
WM: white matter
